# In-hospital mortality is associated with high NT-proBNP level

**DOI:** 10.1371/journal.pone.0207118

**Published:** 2018-11-08

**Authors:** Malik Benmachiche, Pedro Marques-Vidal, Gérard Waeber, Marie Méan

**Affiliations:** Department of Internal Medicine, Lausanne University Hospital, Lausanne, Switzerland; Scuola Superiore Sant'Anna, ITALY

## Abstract

**Objective:**

To compare in-hospital mortality in unselected adult patients according to N-terminal pro-brain natriuretic peptide (NT-proBNP) levels.

**Method:**

Retrospective study including 3833 adult patients (median age 72 years, 45% women) hospitalized between January 2013 and April 2015 in a Swiss university hospital, with at least one NT-proBNP level measurement during hospitalization. Patients were categorized in quintiles regarding their highest NT-proBNP level. In-hospital mortality and length of stay (LOS) were compared between the highest and the other quintiles.

**Results:**

In-hospital mortality rate and LOS (average±standard deviation) were higher in the fifth quintile than in the others (6.5% vs 20.3%, and 20.8±24.0 vs. 14.9±26.5 days respectively, both p<0.001). After multivariate adjustment on age, gender, principal diagnoses, stage 5 renal failure and type of management, patients in the fifth quintile had a hazard ratio [95% confidence interval] of 1.97 [1.57–2.46] for in-hospital mortality and an adjusted LOS (average±standard error) of 20.4±1.0 vs. 14.9±0.5 days for the other quintiles (p<0.001). Further stratification on the main diagnosis at discharge led to similar findings.

**Conclusion:**

Patients with high levels of NT-proBNP are at higher risk of in-hospital mortality and longer LOS, regardless of their clinical characteristics. NT-proBNP level can be a helpful tool for predicting in-hospital patient outcome in unselected adult patients.

## Introduction

B-type natriuretic peptide (BNP) is secreted by cardiomyocites in response to cardiac wall stress and fluid overload to regulate the fluid balance [1,2]. It is synthesized in cardiomyocites as a precursor protein (preproBNP), which is processed to form a propeptide, proBNP. ProBNP is cleaved in the Golgi apparatus of the cardiomyocite, to form a biologically active peptide, BNP, and an inactive N-terminal peptide, NT-proBNP. In case of greatly increased production, such as heart failure, part of the intact proBNP is secreted directly in the circulation, where it can be cleaved by plasmatic corin [3,4]. BNP or NT-proBNP levels are commonly used to diagnose acute heart failure [5] and to monitor the response to treatment [6].

In patients with heart failure, a high BNP level has been shown to be an independent predictor of in-hospital mortality, hospital readmission, lower life expectancy and stroke [7–13]. A high BNP level is also related to prolonged hospital length of stay (LOS) in patients with heart failure [14] or myocardial infarction [15] and in patients undergoing cardiac surgery [16,17]. The ADHERE study, a cohort study of 48’000 patients hospitalized with heart failure, showed that BNP levels >1730 pg/ml were associated with an in-hospital mortality of 6%, versus 1.9% for BNP levels <430 pg/ml [18]. Higher NT-proBNP levels were also associated with higher mortality of patients with pneumonia [19,20] or stroke [21]. In patients with end-stage renal disease, NT-proBNP values >12’200 pg/ml were associated with a 314 days mortality of 68%, three times higher than levels <12’200 pg/ml [22]. Hence, elevated (NT-pro) BNP levels might be useful to predict in-hospital mortality among a wide range of clinical conditions. Indeed, NT-proBNP can increase not only in cardiovascular diseases, but also in several endocrine, metabolic, pulmonary, immunological, infective, and hepatic disorders [23]. Still, most studies focused on specific conditions and not on a multidisciplinary, multipathology setting.

Therefore, we conducted a retrospective study to compare the in-hospital mortality and length of stay of unselected hospitalized patients according to their NT-proBNP levels.

## Materials and methods

### Patient selection

This is a retrospective study conducted at the Lausanne university hospital (CHUV), Switzerland. The target population was all adult patients hospitalized for at least one day between January 2013 and April 2015. Inclusion criteria were: 1) consent for further use of their clinical data and 2) at least one NT-proBNP level measurement during hospitalization. In the case of patients with multiple hospitalizations during the study period, only the last one was considered.

### Ethical considerations and data protection

Since January 2013, all patients hospitalized in the CHUV are asked for general consent that allows future use of medical records and blood tests performed during their hospitalization. The study was specifically approved by the ethics committee (Commission cantonale d’éthique de la recherche sur l’être humain, May 6 2015, ref. CER-VD 209/15) and no further specific consent from the patients was required as only already available administrative data was used. Data was extracted by a dedicated team using the inclusion criteria defined previously and was anonymized before being provided to the investigators. Thus, it was not possible to assess how many patients were excluded.

### Data collection

We collected information regarding age, gender, renal function, total LOS, vital status, type of admission (emergency or not), hospital ward where the NT-proBNP was measured and main diagnosis at discharge. Renal function was based on the glomerular filtration rate (GFR) estimated through the MDRD method, with stage 5 defined as a GFR < 15 ml/min [24]. Main diagnosis at discharge was extracted from the codes of the International Classification of Diseases, 10^th^ revision (ICD-10) and categorized as follows: heart failure (HF, ICD-10 code I50); other heart disease (code I10-I52 except I50); pneumonia (codes J12-J18); chronic pulmonary obstructive disease (COPD, codes J42-J44) and all other diagnosis (all other codes).

NT-proBNP measurements were performed using electrochemiluminescence “ECLIA” method in a Cobas 8000 (Roche Diagnostics, Switzerland), using the proBNP II kit from the same company (inter-batch CV: 4.0%). Patients were categorized in quintiles according to their highest NT-proBNP value during the hospitalization. The NT-proBNP levels defining the different quintiles were: 5–243; 244–819; 820–2271; 2272–6095 and ≥6096 ng/L for the first to the fifth quintile, respectively.

### Outcomes

In-hospital all-cause mortality and LOS were the primary and secondary outcome, respectively. Subgroup analyses comparing in-hospital mortality and LOS between the fifth and the other quintiles of NT-proBNP were also performed after stratifying on the main diagnosis at discharge.

### Statistical analyses

Statistical analysis was conducted using Stata version 14.0 for windows (Stata Corp, College Station, TX, USA). Analyses were performed comparing the last to the other quintiles as the most of the multivariable analyses using individual quintiles could not be performed due to the small number of events within each quintile. Hence, analyses using individual quintiles are provided as supplemental files.

For in-hospital mortality, results are expressed as number of deaths and percentage (bivariate) or as multivariable-adjusted hazard ratio and 95% confidence interval. Between-group comparisons were performed using chi-square or Fisher’s exact test (bivariate) or Cox regression (multivariable). For LOS, results are expressed as average±standard deviation (bivariate) or as multivariable-adjusted average±standard error. Between-group comparisons were performed using student’s t-test on log-transformed values (bivariate) or analysis of variance (multivariable). Multivariable analysis was adjusted for age (continuous), gender, principal diagnoses (HF, other heart disease, pneumonia, COPD and other), stage 5 renal failure (yes/no), hospital ward (medicine, surgery, intensive care) and stay in emergency room (yes/no). Test for trend regarding individual quintiles was performed using the command **contrast p.** after the multivariable analyses. Statistical significance was considered for a two-sided test p<0.05.

Sensitivity analyses were performed by 1) stratifying the analysis according to the main diagnosis at discharge and 2) comparing in-hospital mortality between all quintiles of NT-proBNP.

## Results

### Sample characteristics

**Fig 1:**
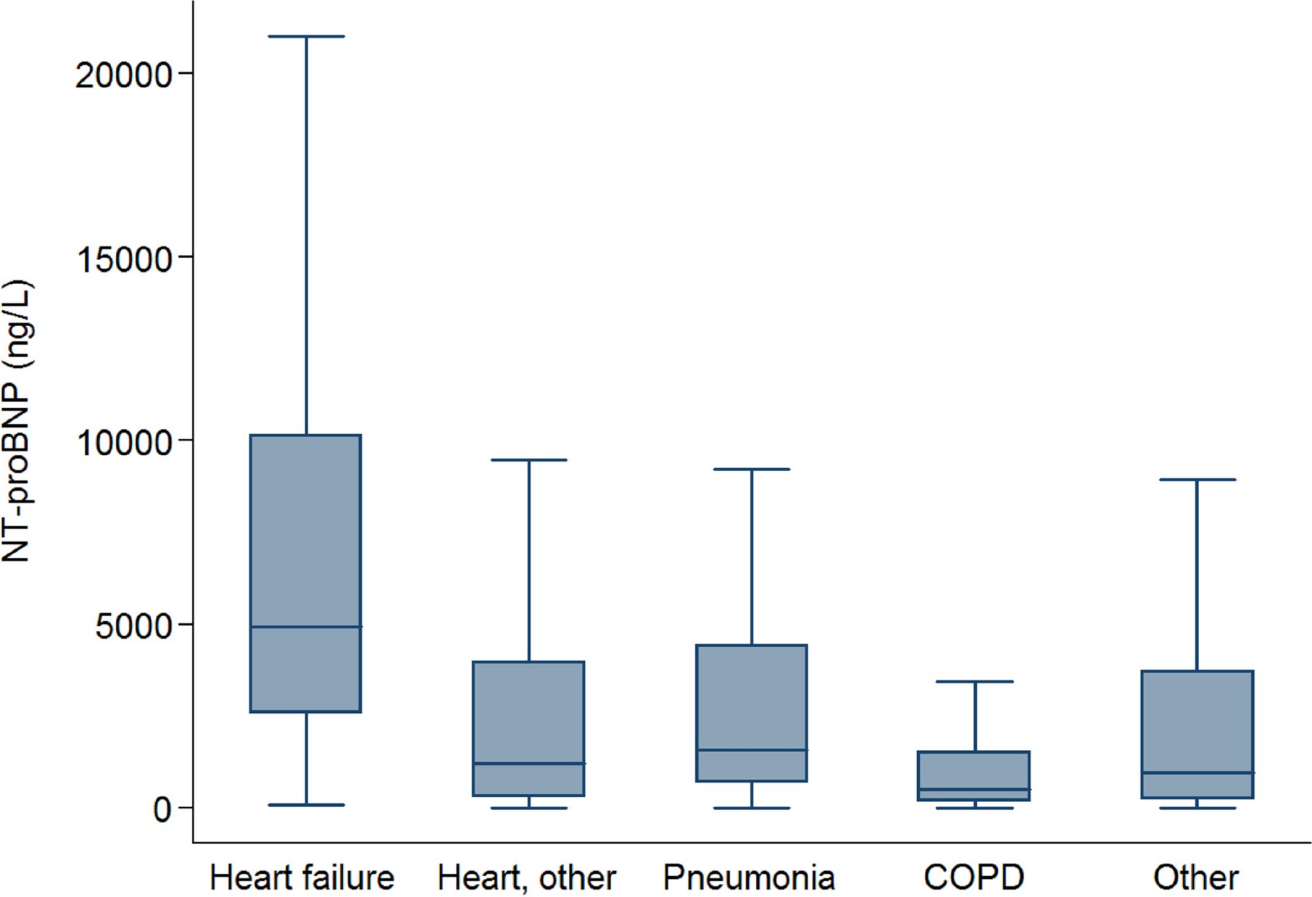
Median and interquartile range values of NT-proBNP according to diagnosis groups.

Overall, 3833 patients were included: 474 (12.4%) had a principal diagnosis of HF, 851 (22.2%) had another heart disease, 229 (6.0%) had pneumonia, 102 (2.7%) COPD and 2177 (56.8%) had another diagnosis. The characteristics of the patients in the fifth and in the other quintiles of NT-proBNP are summarized in **Table 1**. Patients in the fifth quintile were older, had more frequently a main diagnosis of heart failure and of stage 5 renal failure than patients in the other quintiles. Similar findings were observed when the analysis was expanded to individual quintiles (**Table A in S1 File**). Distribution of the NT-proBNP values in each diagnosis group are shown in **Fig 1**. The frequencies of the principal diagnoses of the patients in the group “other” are shown in **Table B in S1 File**.

**Table 1:** Patients’ clinical characteristics according to NT-proBNP quintiles, Lausanne university hospital, 2013–2015.

	All (n = 3833)	First to fourth quintile (n = 3068)	Fifth quintile (n = 765)	p-value
**Age, years**	71.9 ± 15.7	70.3 ± 16	78.3 ± 12.8	<0.001
**Female gender (%)**	1726 (45.0)	1368 (44.6)	358 (46.8)	0.272
**Principal diagnosis (%)**				<0.001
** Heart failure**	474 (12.4)	275 (9.0)	199 (26.0)	
** Other heart disease**	851 (22.2)	712 (23.2)	139 (18.2)	
** Pneumonia**	229 (6.0)	185 (6.0)	44 (5.8)	
** COPD**	102 (2.7)	94 (3.1)	8 (1.1)	
** Other**	2177 (56.8)	1802 (58.7)	375 (49.0)	
**Stage 5 renal failure (%)**	83 (2.2)	25 (0.8)	58 (7.6)	<0.001
**Hospital ward (%)**				<0.001
** Medical**	3061 (79.9)	2479 (80.8)	582 (76.1)	
** Surgery**	411 (10.7)	334 (10.9)	77 (10.1)	
** Intensive care**	361 (9.4)	255 (8.3)	106 (13.9)	

Results are expressed as number of patients (percentage) or as average ± standard deviation. Between-group comparisons performed using chi-square for categorical variables and student’s t-test for continuous variables.

### In-hospital mortality and length of stay, all patients

**Fig 2:**
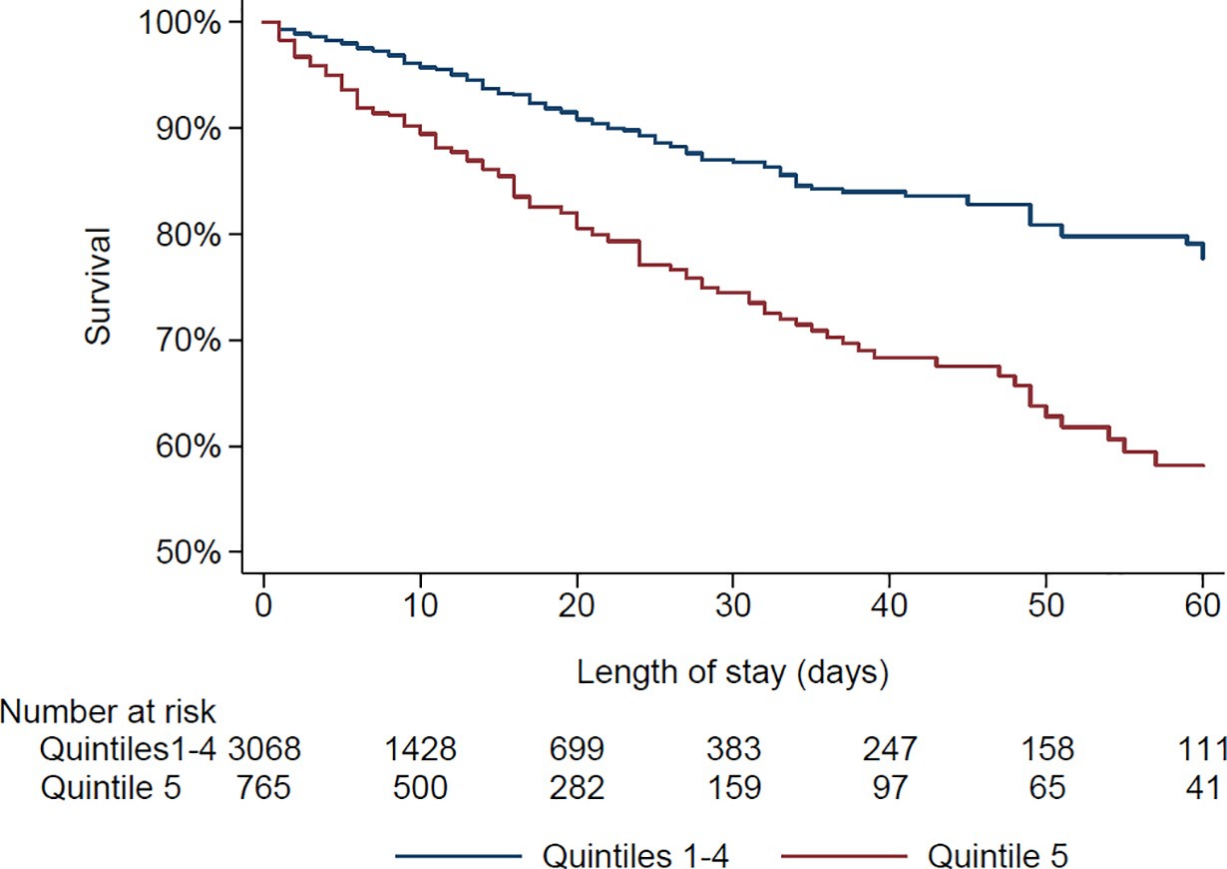
Kaplan-Meyer survival curves according to NT-proBNP level.

Three hundred and fifty-three patients (9.2%) died during the hospitalization. The bivariate and multivariable-adjusted results regarding the association between NT-proBNP quintiles and in-hospital mortality are summarized in **Table 2**. Patients in the fifth quintile of NT-proBNP had higher in-hospital mortality than patients in the other quintile s, and this difference persisted after multivariable adjustment (**Table 2, Fig 2**). At 10 days, the survival rate of patients of the fifth quintile who were still hospitalized was 90% (95% confidence interval: 87–92), vs. 96% (95–97) for the other quintiles. Considering patients in the fifth quintile as being at risk of dying had a sensitivity of 44% (95% confidence interval: 39–49), a specificity of 83% (81–84), a positive predictive value of 20% (18–23) and a negative predictive value of 94% (93–95). When comparing individual quintiles, a positive trend with mortality was found (multivariable-adjusted test for trend: p<0.001, **S1 Fig and Table C in S1 File**).

**Table 2:** Effect of NT-proBNP levels on in-hospital mortality and length of stay, Lausanne university hospital, 2013–2015.

	First to fourth quintile (n = 3068)	Fifth quintile (n = 765)	p-value
In-hospital mortality ^1^			
Bivariate	198 (6.5)	155 (20.3)	<0.001
Multivariable-adjusted ^3^	1 (ref)	1.97 (1.57–2.46)	<0.001
Length of stay (days) ^2^			
Bivariate	14.9 ± 26.5	20.8 ± 24.0	<0.001
Multivariable-adjusted ^3^	14.9 ± 0.5	20.4 ± 1.0	<0.001

^1^ expressed as number of patients (percentage)

^2^ comparisons performed on log-transformed data

^3^ adjusted for age (continuous), gender, principal diagnoses (heart failure, other heart disease, pneumonia, COPD and other), stage 5 renal failure (yes/no), hospital ward (medicine, surgery, intensive care) and stay in emergency room (yes/no). For in-hospital mortality, results are expressed as number of deaths and percentage (bivariate) or Cox regression (multivariable). For length of stay, results are expressed as average±standard deviation (bivariate) or as multivariable-adjusted average±standard error. Between-group comparisons were performed using student’s t-test (bivariate) or analysis of variance (multivariable).

Overall, median length of stay was 10 days (interquartile range: 3–20). The bivariate and multivariable-adjusted results regarding the association between NT-proBNP quintiles and LOS are summarized in **Table 2**. Patients in the fifth quintile of NT-proBNP had longer LOS than patients in the other quintiles, and this difference persisted after multivariable adjustment (**Table 2**).

### In-hospital mortality and length of stay, stratified by diagnosis at discharge

The in-hospital mortality and LOS according to NT-proBNP quintiles and stratified by main diagnosis at discharge is summarized in **Table 3**. In the HF and other heart disease groups, patients in the fifth quintile of NT-proBNP had higher in-hospital mortality on bivariate analysis, but the association was no longer significant on multivariable analysis. In the pneumonia group, mortality was similar in the two groups. In the COPD group, there was only one death in each group. In the other diagnosis group, patients in the fifth quintile of NT-proBNP had doubled in-hospital mortality compared to the other quintiles, and this association persisted after multivariable analysis (**Table 3**). Finally, patients in the fifth quintile of NT-proBNP had longer LOS than patients in the other quintiles in all groups except COPD (**Table 3**). Subgroup analysis for the most frequent diagnoses included in the group “other” are shown in **Table D in S1 File**. Mortality was higher for patients in the highest quintile of proBNP with several other main diagnoses such as infectious diseases (HR 5.41, CI 2.05–14.26), neoplasms (HR 2.57, CI 1.40–4.74) and trauma (HR 4.83, CI 1.71–13.68). Overall, we found a tendency to a higher in-hospital mortality and a longer LOS for patients in the fifth quintile with most of the subdiagnoses of the “other” group. Similar findings were obtained when the analysis was expanded to individual quintiles, with an increase in LOS and in-hospital mortality from the first to the fifth quintile, although some associations could not be assessed due to small sample sizes and/or absence of events (**Table E in S1 File**).

**Table 3:** Subgroup analysis of the effect of NT-proBNP levels on in-hospital mortality and length of stay, Lausanne university hospital, 2013–2015.

	First to fourth quintile	Fifth quintile	p-value
**Heart failure**	(n = 275)	(n = 199)	
In-hospital mortality ^1^			
Bivariate	18 (6.6)	27 (13.6)	0.010
Multivariable-adjusted ^3^	1 (ref.)	1.48 (0.80–2.74)	0.217
Length of stay (days) ^2^			
Bivariate	14.4 ± 23.1	19.0 ± 24.7	0.002
Multivariable-adjusted ^3^	14.3 ± 1.4	19.1 ± 1.7	0.002
**Other heart disease**	(n = 712)	(n = 139)	
In-hospital mortality ^1^			
Bivariate	24 (3.4)	19 (13.7)	<0.001
Multivariable-adjusted ^3^	1 (ref)	1.77 (0.93–3.37)	0.082
Length of stay (days) ^2^			
Bivariate	9.0 ± 10.7	20.6 ± 25.8	<0.001
Multivariable-adjusted ^3^	9.2 ± 0.5	19.2 ± 1.2	<0.001
**Pneumonia**	(n = 185)	(n = 44)	
In-hospital mortality ^1^			
Bivariate	12 (6.5)	5 (11.4)	0.267
Multivariable-adjusted ^3^	1 (ref.)	0.82 (0.23–2.95)	0.762
Length of stay (days) ^2^			
Bivariate	10.0 ± 10.6	15.6 ± 14.2	0.002
Multivariable-adjusted ^3^	9.9 ± 0.8	16.3 ± 1.7	<0.001
**COPD**	(n = 94)	(n = 8)	
In-hospital mortality ^1^			
Bivariate	1 (1.1)	1 (12.5)	0.151
Multivariable-adjusted ^3^	NA	NA	
Length of stay (days) ^2^			
Bivariate	11 ± 18.2	10.9 ± 11.9	0.801
Multivariable-adjusted ^3^	NA	NA	
**Other diagnosis**	(n = 1802)	(n = 375)	
In-hospital mortality ^1^			
Bivariate	143 (7.9)	103 (27.5)	<0.001
Multivariable-adjusted ^3^	1 (ref.)	2.20 (1.68–2.87)	<0.001
Length of stay (days) ^2^			
Bivariate	17.9 ± 31.9	22.7 ± 24.0	<0.001
Multivariable-adjusted ^3^	18.0 ± 0.7	22.2 ± 1.6	<0.001

^1^ expressed as number of patients (percentage)

^2^ comparisons performed on log-transformed data

^3^ adjusted for age (continuous), gender, principal diagnoses (heart failure, other heart disease, pneumonia, COPD and other), stage 5 renal failure (yes/no), hospital ward (medicine, surgery, intensive care) and stay in emergency room (yes/no). For in-hospital mortality, results are expressed as rate (bivariate) or as multivariable-adjusted hazard ratio and (95% confidence interval). Between-group comparisons were performed using chi-square (bivariate) or Cox regression (multivariable). For length of stay, results are expressed as average±standard deviation (bivariate) or as multivariable-adjusted average±standard error. Between-group comparisons were performed using student’s t-test (bivariate) or analysis of variance (multivariable).

## Discussion

In an unselected sample of hospitalized patients, elevated NT-proBNP was associated with increased in-hospital mortality and LOS.

### In-hospital mortality and NT-proBNP levels

Patients with NT-proBNP levels ≥6096 ng/l had double in-hospital mortality and a 5-day longer LOS compared to patients with lower values. Comparison with the literature is limited by the fact that previous studies focused on patients with HF or a single pathology (pneumonia, stroke). Still, these studies also reported that a high NT-proBNP level more than doubled the risk of dying. A Taiwanese single-center study including 269 patients with HF and using a cut-off of 8100 pg/ml reported an Odds-ratio (OR) of 6.65 for in-hospital mortality [25]. Similarly, an international study including 1256 patients with HF and using a cut-off value of 5180 pg/ml found an OR of 5.2 for 76-day mortality [26]. A major issue when comparing results of the literature is the varying cut-offs used to define high NT-proBNP levels. Some studies used cut-off levels which are diagnostic for heart failure (NT-proBNP > 450 pg/ml for patients < 50 years old, > 900 pg/ml for patients between 50 and 75 years old, > 1800 pg/ml for patients > 75 years old) [12,27]. It would be of interest that a consensus is reached regarding thresholds to define high values.

Several studies included NT-proBNP levels in the development of risk scores to predict in-hospital mortality in patients with HF [25,28,29] or as a risk stratification tool for acute exacerbations in patients with COPD [30]. Based on our findings, it is likely that NT-proBNP levels could be used to predict in-hospital mortality in patients with a wider range of pathologies. Still, the diagnostic accuracy of NT-proBNP levels ≥6096 ng/L was rather low (sensitivity 44%, specificity 83%). Further studies are needed to find an adequate threshold which will allow predicting worse outcomes with sufficient reliability to aid decision making and identification of patients who would possibly benefit from palliative care.

### Length of stay and NT-pro-BNP levels

In our study, patients with NT-proBNP levels ≥6096 ng/l (fifth quintile) had a 5-day longer LOS compared to patients with lower values, and these results persisted after adjustment for age, gender, principal diagnosis, stage 5 renal failure, hospital ward and stay in emergency room. This difference was also reported by a study focusing on hospitalizations for acute HF, where patients with BNP levels >1500 pg/ml had a 5-day longer LOS than patients with BNP levels <500 pg/ml [31]. We hereby show similar results among unselected hospitalized patients. This finding might have an important impact on health care cost.

### Subgroup analyses

In the group of patients devoid of pneumonia, COPD or heart-related diseases as main diagnosis, NT-proBNP levels ≥6096 ng/L were associated with a two-fold increased risk of in-hospital mortality. Mortality was higher with most diagnoses in this group, including neoplasms, infectious diseases and trauma. We found no study focusing on this population. A possible explanation is that patients with high NT-proBNP levels might also present with HF or other high-NT-proBNP related comorbidities, thus worsening their prognosis [32]. Still, as comorbidities were not extracted from the database, this hypothesis should be confirmed in future studies.

In patients with HF or other heart diseases, NT-proBNP levels ≥6096 ng/L were also associated with higher mortality and prolonged length of stay, although some associations were not statistically significant. These findings are in agreement with the literature [18,25,28], and the lack of statistical significance might due to the relatively small number of patients. The same applies for patients with pneumonia, where a significant association was found with prolonged LOS but not with in-hospital mortality. Indeed, our sample size of patients with pneumonia (n = 229) was smaller than a previous study (n = 341) focusing on patients with pneumonia, where deceased patients had higher NT-proBNP values than survivors (median 4882 vs. 1133 pg/ml respectively) [20]. The higher mortality among cancer patients could be due either to the presence of HF as a comorbidity, or the cardiotoxic effect of some anticancer drugs such as anthracyclines [33], or both. Indeed, high levels of NT-proBNP have been suggested to be a useful marker for risk assessment in patients treated with chemotherapy [34]. Overall, our results suggest that high NT-proBNP levels are associated with increased in-hospital mortality and LOS in a wider range of pathologies than previously thought.

### Strengths and limitations

As far as we know, this is the first study to compare in-hospital mortality and LOS in unselected hospitalized patients according to their NT-proBNP levels. This study also has limitations. First, because of its retrospective design, only patients with a NT-proBNP measurement were included. This could lead to a selection bias, as patients suspected of HF might be overrepresented. Second, due to the use of ICD-10 codes and no access to echocardiographic data, it was not possible to distinguish between heart failure with preserved ejection fraction and heart failure with reduced ejection fraction. Third, it was not possible to assess the comorbidities or secondary diagnoses of the patients, so the presence of HF or other conditions known to influence NT-proBNP as a comorbidity could not be confirmed. NT-proBNP levels can be influenced directly by renal failure and by obesity [35], and renal failure and HF are potentially directly related [36]. Even though we adjusted our results for end-stage renal failure, other stages of renal insufficiency were not available. Multimorbidity, socio-economic and socio-demographic factors are known to influence hospital stay and mortality [37,38]. As these data were not available, there could be confounding factors that would make our results spurious. However, previous studies adjusted for comorbidities and socio-economic status, made on all-coming outpatient general populations, showed that NT-proBNP was an independent predictor of mortality even for patients devoid of heart failure [39–41]. Our study shows similar results, but with in-hospital patients.

Fourth, we could not adjudicate mortality cause because of the retrospective design of our study. Fifth, this was a monocentric study conducted at a university hospital, which may limit the generalizability of the results. Still, Lausanne University Hospital acts both as university and a general hospital receiving patients from all over Vaud canton. Hence, although our results might not be generalizable to the total population of hospitalized patients, still, they indicate that when a NT-proBNP measurement is prescribed and the value is ≥6096 ng/l, the likelihood of in-hospital death almost doubles. Finally, the relatively small sample size of several subgroups led to reduced statistical power and precluded the detection of statistically significant associations; still, the magnitude of the associations was in line with the literature.

## Conclusion

Patients with high levels of NT-proBNP are at higher risk of in-hospital mortality and present longer LOS, regardless of their clinical characteristics. NT-proBNP level might be a helpful tool for predicting in-hospital outcome in a wider range of pathologies than previously thought.

## Supporting information

S1 File. Supporting tables A-E (DOCX)

S1 Fig Kaplan-Meyer survival curves according to quintiles of NT-proBNP level. (TIF)
