# Supplementary material for: In-hospital mortality is associated with high NT-proBNP level
Source: PLoS One. 2018 Nov 8;13(11):e0207118. doi: 10.1371/journal.pone.0207118 (PMC6224094; doi:10.1371/journal.pone.0207118)
Supplement: S1 File. Supporting tables A-E — (DOCX) [file pone.0207118.s001.docx]

**Table A: Patients’ clinical characteristics according to NT-proBNP quintiles, Lausanne university hospital, 2013-2015.**

|  | First | Second | Third | Fourth | Fifth | p-value |
| --- | --- | --- | --- | --- | --- | --- |
|  | 767 | 767 | 766 | 768 | 765 |  |
| Age, years | 59.7 ± 16.1 | 71.5 ± 13.9 | 73.3 ± 14.8 | 76.6 ± 13.9 | 78.3 ± 12.8 | <0.001 |
| Female gender (%) | 289 (37.7) | 356 (46.4) | 353 (46.1) | 370 (48.2) | 358 (46.8) | <0.001 |
| Principal diagnosis (%) |  |  |  |  |  | <0.001 |
| Heart failure | 2 (0.3) | 19 (2.5) | 77 (10.1) | 177 (23.1) | 199 (26.0) |  |
| Other heart disease | 183 (23.9) | 185 (24.1) | 172 (22.5) | 172 (22.4) | 139 (18.2) |  |
| Pneumonia | 20 (2.6) | 54 (7.0) | 64 (8.4) | 47 (6.1) | 44 (5.8) |  |
| COPD | 30 (3.9) | 29 (3.8) | 24 (3.1) | 11 (1.4) | 8 (1.1) |  |
| Cancer | 49 (6.4) | 56 (7.3) | 63 (8.2) | 41 (5.3) | 38 (5.0) |  |
| Other | 483 (63.0) | 424 (55.3) | 366 (47.8) | 320 (41.7) | 337 (44.1) |  |
| Stage 5 renal failure (%) | 1 (0.1) | 6 (0.8) | 3 (0.4) | 15 (2.0) | 58 (7.6) | <0.001 § |
| Hospital ward (%) |  |  |  |  |  | <0.001 |
| Medical | 651 (84.9) | 634 (82.7) | 608 (79.4) | 586 (76.3) | 582 (76.1) |  |
| Surgery | 62 (8.1) | 81 (10.6) | 90 (11.8) | 101 (13.2) | 77 (10.1) |  |
| Intensive care | 54 (7.0) | 52 (6.8) | 68 (8.9) | 81 (10.6) | 106 (13.9) |  |

Results are expressed as number of patients (percentage) or as average ± standard deviation. Between-group comparisons performed using chi-square or Fisher’s exact test (§) for categorical variables and analysis of variance for continuous variables.

**Table B. Distribution of the principal diagnoses of the group « other »**

| **Diagnosis** | **ICD-10 codes** | **N (% of total)** |
| --- | --- | --- |
| Infectious diseases | A00-B99 | 145 (3.8) |
| Neoplasms | C00-D48 | 233 (6.1) |
| Hematologic disorders | D50-D89 | 23 (0.6) |
| Endocrine, nutritional and metabolic diseases | E00-E99 | 36 (0.9) |
| Dementia | F00-F03 | 2 (0.1) |
| Disorders of nervous system other than stroke | G00-G99 | 54 (1.4) |
| Cerebrovascular diseases | I60-I69 | 81 (2.1) |
| Vascular diseases | I70-I99 | 110 (2.9) |
| Other respiratory diseases | J00-J11; J19-J41; J45-J99 | 280 (7.3) |
| Diseases of the digestive system | K00-K99 | 127 (3.3) |
| Diseases of the musculoskeletal system and connective tissue | M00-M99 | 111 (2.9) |
| Trauma | S00-S99; T01-T35; T66-T88 | 183 (4.8) |
| Toxicology / poisoning | T36-T65 | 4 (0.1) |
| Mental and behavioral disorders, otorhinolaryngology, urology, gynecology, dermatology, congenital disease, factors influencing health status | F04-F99; H00-H99; N00-N99; Q00-Q99; Z00-Z99 | 189 (4.9) |
| Symptoms, signs and abnormal clinical and laboratory findings, not elsewhere classified | R00-R99 | 229 (6.0) |
| No ICD-10 codes | - | 370 (9.6) |
| Total |  | 2177 (56.8) |

**Table C: in-hospital mortality and length of stay according to quintiles of NT-proBNP, Lausanne university hospital, 2013-2015.**

|  | First | Second | Third | Fourth | Fifth | p-value |
| --- | --- | --- | --- | --- | --- | --- |
|  | 767 | 767 | 766 | 768 | 765 |  |
| In-hospital mortality ^1^ |  |  |  |  |  |  |
| Bivariate | 20 (2.6) | 37 (4.8) | 64 (8.4) | 77 (10) | 155 (20.3) | <0.001 |
| Multivariable-adjusted ^3^ | 1 (ref) | 1.25 (0.72 - 2.17) | 1.68 (1.01 - 2.80) | 1.97 (1.18 - 3.28) | 3.20 (1.96 - 5.22) | <0.001 § |
| Length of stay (days) ^2^ |  |  |  |  |  |  |
| Bivariate | 11.2 ± 19.2 | 13.2 ± 19.1 | 16.9 ± 24.5 | 18.1 ± 38.1 | 20.8 ± 24 | <0.001 |
| Multivariable-adjusted ^3^ | 9.9 ± 1.0 | 13.2 ± 0.9 | 17.0 ± 0.9 | 18.8 ± 0.9 | 21.3 ± 1.0 | <0.001 § |

^1^ expressed as number of patients (percentage); ^2^ comparisons performed on log-transformed data; ^3^ adjusted for age (continuous), gender, principal diagnoses (heart failure, other heart disease, pneumonia, COPD and other), stage 5 renal failure (yes/no), hospital ward (medicine, surgery, intensive care) and stay in emergency room (yes/no). §, p-value for test of trend.

For in-hospital mortality, results are expressed as number of patients (percentage) or as multivariable-adjusted hazard ratio and (95% confidence interval). Between-group comparisons were performed using chi-square (bivariate) or Cox regression (multivariable). For length of stay, results are expressed as average±standard deviation (bivariate) or as multivariable-adjusted average±standard error. Between-group comparisons were performed using analysis of variance (bivariate and multivariable).

**Table D. Subgroup analysis of the effect of NT-proBNP levels on in-hospital mortality and length of stay for the patients in the “other diagnosis” group, Lausanne university hospital, 2013-2015.**

|  | First to fourth quintile | Last quintile | p-value |
| --- | --- | --- | --- |
| Infectious diseases |  |  |  |
| In-hospital mortality ^1^ | N=95 | N=50 |  |
| Bivariate | 10 (10.5) | 15 (30.0) | 0.003 |
| Multivariable-adjusted ^3^ | 1 (ref.) | 5.41 (2.05 - 14.26) | 0.001 |
| Length of stay (days) ^2^ | N=95 | N=50 |  |
| Bivariate | 25.7 ± 25.0 | 17.7 ± 19.6 | 0.008 |
| Multivariable-adjusted ^3^ | 25.0 ± 2.4 | 19.1 ± 3.4 | 0.020 |
| Neoplasms |  |  |  |
| In-hospital mortality ^1^ | N=196 | N=37 |  |
| Bivariate | 33 (16.8) | 19 (51.4) | <0.001 |
| Multivariable-adjusted ^3^ | 1 (ref.) | 2.57 (1.40 - 4.74) | 0.002 |
| Length of stay (days) ^2^ | N=196 | N=37 |  |
| Bivariate | 26.6 ± 21.4 | 30.2 ± 24.4 | 0.706 |
| Multivariable-adjusted ^3^ | 26.9 ± 1.6 | 28.6 ± 3.7 | 0.975 |
| Vascular diseases |  |  |  |
| In-hospital mortality ^1^ | N=79 | N=13 |  |
| Bivariate | 4 (5.1) | 7 (22.6) | 0.011 § |
| Multivariable-adjusted ^3^ | 1 (ref.) | 2.2 (0.57 - 8.54) | 0.254 |
| Length of stay (days) ^2^ | N=79 | N=13 |  |
| Bivariate | 18.2 ± 17.6 | 29.8 ± 22.0 | 0.005 |
| Multivariable-adjusted ^3^ | 18.4 ± 2.2 | 29.1 ± 3.6 | 0.033 |
| Other respiratory diseases |  |  |  |
| In-hospital mortality ^1^ | N=224 | N=56 |  |
| Bivariate | 29 (13.0) | 15 (26.8) | 0.011 |
| Multivariable-adjusted ^3^ | 1 (ref.) | 1.29 (0.67 - 2.5) | 0.445 |
| Length of stay (days) ^2^ | N=224 | N=56 |  |
| Bivariate | 15.8 ± 19.1 | 21.7 ± 26.3 | 0.035 |
| Multivariable-adjusted ^3^ | 15.8 ± 1.3 | 21.8 ± 2.7 | 0.025 |
| Diseases of the digestive system |  |  |  |
| In-hospital mortality ^1^ | N=103 | N=24 |  |
| Bivariate | 10 (9.7) | 9 (37.5) | 0.001 |
| Multivariable-adjusted ^3^ | 1 (ref.) | 2.39 (0.89 - 6.38) | 0.083 |
| Length of stay (days) ^2^ | N=103 | N=24 |  |
| Bivariate | 19.8 ± 20.5 | 29.5 ± 26.5 | 0.262 |
| Multivariable-adjusted ^3^ | 19.6 ± 2.1 | 30.3 ± 4.4 | 0.471 |
| Diseases of the musculoskeletal system and connective tissue |  |  |  |
| In-hospital mortality ^1^ | N=93 | N=18 |  |
| Bivariate | 0 (0) | 1 (5.6) | 0.162 § |
| Multivariable-adjusted ^3^ | NA | NA |  |
| Length of stay (days) ^2^ | N=93 | N=18 |  |
| Bivariate | 21.8 ± 20.3 | 35.1 ± 25.5 | 0.026 |
| Multivariable-adjusted ^3^ | 23.2 ± 2.1 | 27.8 ± 5.1 | 0.178 |
| Trauma |  |  |  |
| In-hospital mortality ^1^ | N=152 | N=31 |  |
| Bivariate | 15 (9.9) | 8 (25.8) | 0.015 |
| Multivariable-adjusted ^3^ | 1 (ref.) | 4.83 (1.71 - 13.68) | 0.003 |
| Length of stay (days) ^2^ | N=152 | N=31 |  |
| Bivariate | 21.1 ± 21.3 | 17.4 ± 10.7 | 0.623 |
| Multivariable-adjusted ^3^ | 20.8 ± 1.6 | 18.8 ± 3.5 | 0.491 |
| Other ✝ |  |  |  |
| In-hospital mortality ^1^ | N=152 | N=31 |  |
| Bivariate | 6 (3.9) | 8 (22.2) | <0.001 |
| Multivariable-adjusted ^3^ | 1 (ref.) | 3.03 (0.68 - 13.6) | 0.148 |
| Length of stay (days) ^2^ | N=152 | N=31 |  |
| Bivariate | 18.4 ± 23.9 | 18.7 ± 16.1 | 0.479 |
| Multivariable-adjusted ^3^ | 18.0 ± 1.9 | 20.4 ± 4.2 | 0.457 |
| Symptoms, signs and abnormal clinical and laboratory findings, not elsewhere classified |  |  |  |
| In-hospital mortality ^1^ | N=152 | N=31 |  |
| Bivariate | 6 (3.1) | 12 (35.3) | <0.001 |
| Multivariable-adjusted ^3^ | 1 (ref.) | 7.24 (2.44 - 21.4) | <0.001 |
| Length of stay (days) ^2^ | N=152 | N=31 |  |
| Bivariate | 8.1 ± 10.3 | 12.7 ± 15.7 | 0.162 |
| Multivariable-adjusted ^3^ | 8.4 ± 0.8 | 11.5 ± 2.0 | 0.557 |
| No ICD-10 code |  |  |  |
| In-hospital mortality ^1^ | N=347 | N=23 |  |
| Bivariate | 2 (0.6) | 1 (4.4) | 0.176 § |
| Multivariable-adjusted ^3^ | 1 (ref.) | 0.29 (0.01 - 12.5) | 0.520 |
| Length of stay (days) ^2^ | N=347 | N=23 |  |
| Bivariate | 15.4 ± 59.5 | 30.4 ± 47.6 | <0.001 |
| Multivariable-adjusted ^3^ | 15.5 ± 2.5 | 29.3 ± 10.0 | 0.011 |

^1^ expressed as number of patients (percentage); ^2^ comparisons performed on log-transformed data; ^3^ adjusted for age (continuous), gender, hospital ward (medicine, surgery, intensive care) and stay in emergency room (yes/no). Due to small sample sizes, adjustment for renal failure could not be performed. For in-hospital mortality, results are expressed as rate (bivariate) or as multivariable-adjusted hazard ratio and (95% confidence interval). Between-group comparisons were performed using chi-square or Fisher’s exact test (§) (bivariate) or Cox regression (multivariable). For length of stay, results are expressed as average±standard deviation (bivariate) or as multivariable-adjusted average±standard error. Between-group comparisons were performed using student’s t-test (bivariate) or analysis of variance (multivariable).

✝ Mental and behavioural disorders, otorhinolaringology, urology, gynecology, dermatology, congenital disease and factors influencing health status. Please consult S1 table for a listing of the different ICD-10 codes used to define each group

**Table E: in-hospital mortality and length of stay according to quintiles of NT-proBNP, stratified by main diagnosis, Lausanne university hospital, 2013-2015.**

|  | First | Second | Third | Fourth | Fifth | p-value |
| --- | --- | --- | --- | --- | --- | --- |
| Heart failure (N) | 2 | 19 | 77 | 177 | 199 |  |
| In-hospital mortality ^1^ |  |  |  |  |  |  |
| Bivariate | 0 (0) | 0 (0) | 6 (7.8) | 12 (6.8) | 27 (13.6) | 0.116 |
| Multivariable-adjusted ^3^ | 1 (ref) | NA | NA | NA | NA |  |
| Length of stay (days) ^2^ |  |  |  |  |  |  |
| Bivariate | 13.5 ± 2.1 | 8.1 ± 9.9 | 15.1 ± 35.5 | 14.8 ± 16.5 | 19.0 ± 24.7 | 0.002 |
| Multivariable-adjusted ^3^ | 13.4 ± 16.5 | 7.7 ± 5.5 | 14.9 ± 2.7 | 14.7 ± 1.8 | 19.2 ± 1.7 | 0.985 § |
| Other heart disease (N) | 183 | 185 | 172 | 172 | 139 |  |
| In-hospital mortality ^1^ |  |  |  |  |  |  |
| Bivariate | 5 (2.7) | 1 (0.5) | 9 (5.2) | 9 (5.2) | 19 (13.7) | <0.001 |
| Multivariable-adjusted ^3^ | 1 (ref) | NA | NA | NA | NA |  |
| Length of stay (days) ^2^ |  |  |  |  |  |  |
| Bivariate | 5.2 ± 7.3 | 7.0 ± 7.4 | 10.3 ± 10.1 | 13.7 ± 14.6 | 20.6 ± 25.8 | <0.001 |
| Multivariable-adjusted ^3^ | 5.5 ± 1.1 | 7.6 ± 1.0 | 10.3 ± 1.0 | 13.4 ± 1.1 | 19.9 ± 1.2 | <0.001 § |
| Pneumonia (N) | 20 | 54 | 64 | 47 | 44 |  |
| In-hospital mortality ^1^ |  |  |  |  |  |  |
| Bivariate | 0 (0) | 5 (9.3) | 3 (4.7) | 4 (8.5) | 5 (11.4) | 0.490 |
| Multivariable-adjusted ^3^ | 1 (ref) | NA | NA | NA | NA |  |
| Length of stay (days) ^2^ |  |  |  |  |  |  |
| Bivariate | 8.6 ± 6.2 | 9.8 ± 9.0 | 10.9 ± 13.4 | 9.9 ± 9.5 | 15.6 ± 14.2 | 0.041 |
| Multivariable-adjusted ^3^ | 6.3 ± 2.5 | 9.2 ± 1.5 | 11.0 ± 1.4 | 10.6 ± 1.6 | 16.4 ± 1.7 | 0.007 § |
| COPD (N) |  |  |  |  |  |  |
| In-hospital mortality ^1^ | 30 | 29 | 24 | 11 | 8 |  |
| Bivariate | 0 (0) | 1 (3.5) | 0 (0) | 0 (0) | 1 (12.5) | 0.116 |
| Multivariable-adjusted ^3^ | 1 (ref) | NA | NA | NA | NA |  |
| Length of stay (days) ^2^ |  |  |  |  |  |  |
| Bivariate | 7.6 ± 10.6 | 15 ± 28.3 | 10.2 ± 11.8 | 11.3 ± 9.6 | 10.9 ± 11.9 | 0.534 |
| Multivariable-adjusted ^3^ | NA | NA | NA | NA | NA |  |
| Other diagnosis (N) | 49 | 56 | 63 | 41 | 38 |  |
| In-hospital mortality ^1^ |  |  |  |  |  |  |
| Bivariate | 2 (4.1) | 11 (19.6) | 10 (15.9) | 11 (26.8) | 19 (50.0) | <0.001 |
| Multivariable-adjusted ^3^ | 1 (ref) | 4.46 (0.97 - 20.4) | 3.53 (0.75 - 16.5) | 6.58 (1.41 - 30.8) | 10.3 (2.27 - 46.5) | 0.002 § |
| Length of stay (days) ^2^ |  |  |  |  |  |  |
| Bivariate | 23.3 ± 19.4 | 26.1 ± 21.0 | 28.1 ± 23.2 | 28.0 ± 20.5 | 30.0 ± 24.1 | 0.652 |
| Multivariable-adjusted ^3^ | 24.1 ± 3.3 | 25.3 ± 2.9 | 28.8 ± 2.7 | 28.2 ± 3.4 | 28.7 ± 3.6 | 0.357 § |

^1^ expressed as number of patients (percentage); ^2^ comparisons performed on log-transformed data; ^3^ adjusted for age (continuous), gender, stage 5 renal failure (yes/no), hospital ward (medicine, surgery, intensive care) and stay in emergency room (yes/no). §, p-value for test of trend; NA, not assessable.

For in-hospital mortality, results are expressed as number of patients (percentage) or as multivariable-adjusted hazard ratio and (95% confidence interval). Between-group comparisons were performed using Fisher’s exact test (bivariate) or Cox regression (multivariable). For length of stay, results are expressed as average±standard deviation (bivariate) or as multivariable-adjusted average±standard error. Between-group comparisons were performed using analysis of variance (bivariate and multivariable).
