# Supplementary figures and images for: In-hospital mortality is associated with high NT-proBNP level
Source: PLoS One. 2018 Nov 8;13(11):e0207118. doi: 10.1371/journal.pone.0207118 (PMC6224094; doi:10.1371/journal.pone.0207118)

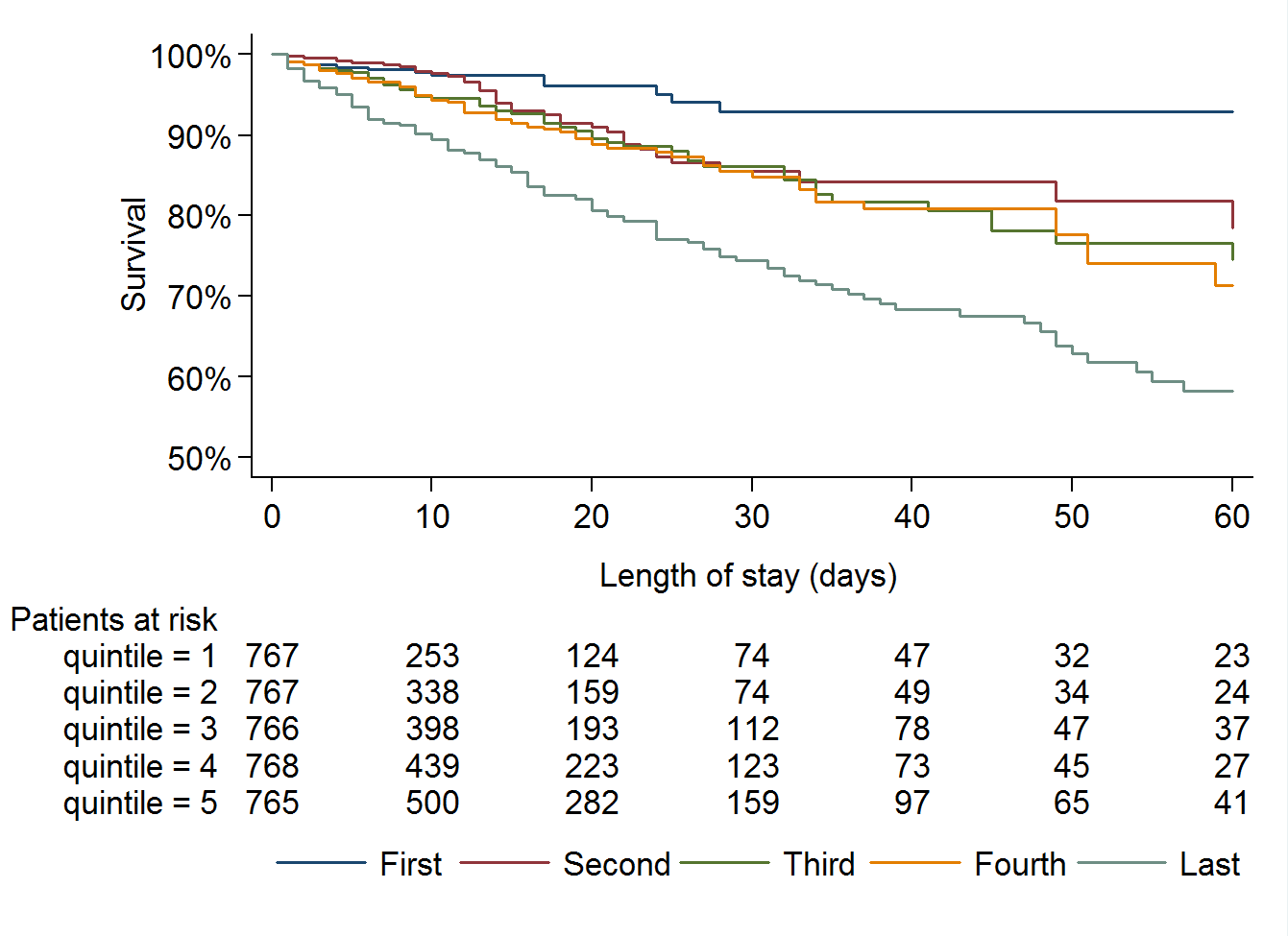

Supplement: S1 Fig — (TIF) [file pone.0207118.s002.tif]
